# Rapid diagnosis of bloodstream infections using a culture-free phenotypic platform

**DOI:** 10.1038/s43856-024-00487-x

**Published:** 2024-04-23

**Authors:** Xuyang Shi, Shivani Sharma, Richard A. Chmielewski, Mario J. Markovic, J. Scott VanEpps, Siu-Tung Yau

**Affiliations:** 1 Department of Electrical and Computer Engineering, Cleveland State University, Cleveland, OH USA; 2 Rapidect Inc., Solon, OH USA; 3 Saint Vincent Charity Medical Center, Cleveland, OH USA; 4 Department of Laboratory Medicine, Saint Vincent Charity Medical Center, Cleveland, OH USA; 5 Department of Emergency Medicine, University of Michigan, Ann Arbor, MI USA; 6 Weil Institute for Critical Care Research and Innovation, University of Michigan, Ann Arbor, MI USA; 7 Biointerfaces Institute, University of Michigan, Ann Arbor, MI USA; 8 The Applied Bioengineering Program, Cleveland State University, Cleveland, OH USA

**Keywords:** Diagnosis, Antimicrobials

## Abstract

**Background:**

Bloodstream infections (BSIs) are a life-threatening acute medical condition and current diagnostics for BSIs suffer from long turnaround time (TAT). Here we show the validation of a rapid detection-analysis platform (RDAP) for the diagnosis of BSIs performed on clinical blood samples

**Methods:**

The validation was performed on a cohort of 59 clinical blood samples, including positive culture samples, which indicated confirmed bloodstream infections, and negative culture samples. The bacteria in the positive culture samples included Gram-positive and Gram-negative pathogenic species. RDAP is based on an electrochemical sandwich immunoassay with voltage-controlled signal amplification, which provides an ultra-low limit of detection (4 CFU/mL), allowing the platform to detect and identify bacteria without requiring culture and perform phenotypic antibiotic susceptibility testing (AST) with only 1–2 h of antibiotic exposure. The preliminary diagnostic performance of RDAP was compared with that of standard commercial diagnostic technologies.

**Results:**

Using a typical clinical microbiology laboratory diagnostic workflow that involved sample culture, agar plating, bacteria identification using matrix-assisted laser desorption ionization time-of-flight (MALDI TOF) mass spectrometry, and AST using MicroScan as a clinical diagnostic reference, RDAP showed diagnostic accuracy of 93.3% and 95.4% for detection-identification and AST, respectively. However, RDAP provided results at least 15 h faster.

**Conclusions:**

This study shows the preliminary feasibility of using RDAP to rapidly diagnose BSIs, including AST. Limitations and potential mitigation strategies for clinical translation of the present RDAP prototype are discussed. The results of this clinical feasibility study indicate an approach to provide near real-time diagnostic information for clinicians to significantly enhance the treatment outcome of BSIs.

## Plain language summary

Effective treatment of bloodstream infections (BSIs), a life-threatening acute medical condition, requires rapid diagnosis. Current diagnostic methods involve culturing the bacteria from the patient’s blood, which requires typically 16–48 h to produce a diagnosis. Here, we demonstrate the feasibility of using a culture-free platform to perform rapid diagnosis of BSIs. We tested the diagnostic platform on a cohort of clinical blood samples. The bacteria contained in the samples covered a representative range of bacteria that cause BSIs. The culture-free platform produced diagnosis in about 15 hours faster than standard commercial diagnostic technologies and  the diagnostic results were in good agreement with that of standard technologies. The results of this study indicate an approach to providing near real-time diagnostic information for clinicians to significantly enhance the treatment outcome of BSIs.

## Abstract (web summary)

Shi et al. describe a phenotypic, culture-free platform that completes the diagnosis of bloodstream infections about 15 hours faster than standard diagnostic technologies. Results from clinical blood samples that demonstrate near real-time diagnostic information suitable for clinicians is provided.

## Introduction

BSI is manifested by a loss of barrier function (e.g., skin or mucosa) that allows bacteria to enter the bloodstream, causing an inflammatory response^1^. BSI remains a formidable medical issue, with over two million cases in the United States annually^2^. At least 44,000 people die each year as a direct result of this medical condition^3^. The burden caused by BSI on the U.S. economy was estimated to be $20 billion in excess of direct healthcare costs in 2013^4^, which ranged from $18,000 to more than $90,000 per case^5^.

The current gold-standard for the diagnosis of BSIs is based on culture and consists of three steps: detection of bacteria (16–48 h), identification (ID) of the species of bacteria (<1 h), and antibiotics susceptibility testing (AST; 16–24 h)^6,7^. Therefore, the total TAT of culture-based diagnosis is 36–72 h. Numerous studies show that timely administration of appropriate antibiotics is critical to improved outcomes of  BSIs and, more specifically, sepsis^8^ and delayed administration of antibiotics leads to increased mortality in BSIs and the development of sepsis^9,10^. Therefore, during this long time of diagnostic uncertainty, broad-spectrum antibiotics are administered to cover the most likely pathogens^11^. However, empiric, broad-spectrum antibiotics lead to under- or over-treatment, severe adverse effects (i.e., renal or hepatic toxicity), morbidity, and mortality, as well as contribute to the development of antibiotic resistance. Further, culture-based diagnosis suffers from unsatisfactory sensitivity and specificity^12^.

It can be argued that the most critical step in the diagnostic workflow is AST because certain strains of bacteria have developed resistance against the antibiotics that were developed to treat them. The prevalence of multiple-drug-resistant organisms (MDROs) is currently poised as one of the greatest threats to public health^13^. Although the bacterial concentration in BSIs can be extremely low (ca. 1–10 CFU/mL^14^), if not rapidly treated, BSIs may progress to sepsis and septic shock^9^. Therefore, there is a critical unmet need to develop strategies that provide rapid and accurate diagnosis of BSI with particular emphasis on AST.

Current AST technologies are largely based on either genotypic or phenotypic approaches. Genotypic technologies such as FilmArray (BioFire) and LightCycler (Roche Diagnostics) rely on the detection of antibiotic resistance genes, whereas phenotypic technologies such as Accelerate Pheno monitor the continuous change in bacterial concentration in the presence of antibiotics. Since many resistance mechanisms are not known at a genotypic level or are not ascribed to single genes, genotypic methods may not provide sufficient diagnostic information for clinical decision-making regarding antibiotic selection^15^. Further, most of the aforementioned commercial AST technologies require positive culture as an input and therefore are subject to the delayed TAT, sensitivity, and specificity issues mentioned above. There are also notable emerging diagnostic methods that have recently been published. Integrated droplet digital detection combines a DNAzyme sensor, droplet microfluidics, and a 3D laser-based particle counter for the detection of bloodstream infections^16^. The detection limit is 1 CFU/mL, and the assay time varies from 90 min to 4 h. However, this method requires diluted blood samples, which require extra time to prepare samples and may introduce inaccuracy. A combined quantitative PCR-based ID-AST assay with sequential detection, ID, and AST of leading bacterial pathogens has been developed^17^. The method features a detection limit of 1 CFU/mL. However, there was significant sample preparation involved, including centrifugation to form bacterial pellets and two 4-h culture-enrichment processes, to achieve this limit of detection.

The realization of our diagnostic platform, RDAP, was evolved from our attempt to use electrostatic means to control charge transport in biological systems. This idea has resulted in our demonstration of the protein transistor^18^ and electrostatic control of cellular metabolic processes^19^. Based on these works, we developed an electrochemical biosensing technique, field effect enzymatic detection (FEED), which features voltage-controlled intrinsic signal amplification^20^. FEED has demonstrated glucose detection at the picomolar level. To utilize the signal amplification feature of FEED to solve health-related issues, we subsequently developed RDAP, a culture-free, phenotypic detection platform^21^ for the diagnosis of bacterial infections. In our first proof-of-concept study, we demonstrated, using contrived samples (*E. coli* spiked in blood), the feasibility of using this platform to perform the three-step diagnostic workflow^21^. The RDAP system is a modified electrochemical sandwich immunoassay platform, whose TAT is ultimately limited only by the sample–antibody incubation time. With the current prototype, we were able to achieve a limit of detection (LOD) of 4 CFU/mL with a TAT of 88 min for detection and ID without culture and 148 min for AST per antibiotic with only 1–2 h of antibiotic exposure. The present article describes our transition from contrived samples to real-world clinical samples and reports the diagnostic performance of RDAP on a cohort of 59 clinical blood samples. The objective is to perform our first clinical comparison of RDAP to current standard microbiology culture testing, including detection and identification (*N* = 32, 16 positive/16 negative) as well as AST (*N* = 27).

In this study, we show the feasibility of using RDAP to rapidly diagnose BSIs, including AST. We show that RDAP is capable of achieving diagnostic accuracy of 93.3% and 95.4% for detection-identification and AST, respectively. However, RDAP provides results at least 15 h faster. Limitations and potential mitigation strategies for clinical translation of the present RDAP prototype are discussed. The results of this study indicate an approach to provide near real-time diagnostic information for clinicians to significantly enhance the treatment outcome of BSIs.

## Methods

### Reagents

#### Clinical samples

**Fig. 1:**
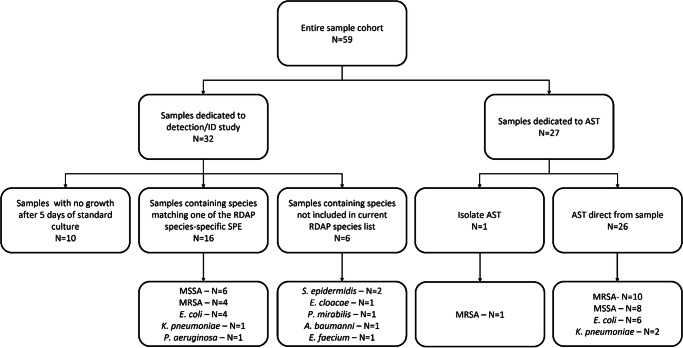
Distribution of clinical samples. The diagram shows the way the clinical samples were used for different measurements within this study.

Fifty-nine (59) blood samples from vacutainer tubes containing sodium citrate as the anticoagulant were obtained from excess de-identified blood samples that were concurrently undergoing culture in standard blood culture bottles during usual clinical care under IRB (No. 580) from the Clinical Microbiology Laboratory at Saint Vincent Charity Medical Center (SVCMC) in Cleveland, OH. This IRB did not require the informed consent process because the samples were de-identified. The blood culture bottles were used to perform detection, identification, and AST using standard clinical microbiology laboratory methods (see “Standard laboratory diagnosis procedure” below). The excess  samples were stored at 4 °C prior to measurements on RDAP, which yielded consistent results. The 59 samples consisted of positive and negative blood cultures. Because the clinical samples used in this study were excess samples, the volume of each sample was not enough to be used for both AST and ID. The excess samples were used for different measurements within this study, according to Fig. 1.

#### Antibodies

Commercial antibodies were used to form sandwich immune complexes on the working electrode of screen-printed electrodes (SPEs). For *Escherichia coli* (*E. coli*) detection, *E. coli* serotype O/K polyclonal antibody (Thermo Fisher Scientific, PA1-7213) was used as the capture antibody (Ab^c^), and horseradish peroxidase (HRP)-coupled *E. coli* serotype O/K polyclonal antibody (Thermo Fisher Scientific, PA1-73030) was used as the detection antibody (Ab^d^). For *Klebsiella pneumoniae* (*K. pneumoniae*) detection, *K. pneumoniae* Rb pAb antibody (Abcam, ab21117) was used as Ab^c^, and HRP-coupled *K. pneumoniae* antibody (Invitrogen, PA1-73176) was used as Ab^d^. For *Pseudomonas aeruginosa* (*P. aeruginosa*) detection, *Pseudomonas* antibody (Invitrogen, MA1-83430) was used as Ab^c^, and HRP-coupled *Pseudomonas* antibody (Invitrogen, PA1-73118) was used as Ab^d^. For *Neisseria gonorrhoeae* (*N. gonorrhoeae*) detection, *N. gonorrhoeae* antibody (Invitrogen, MA1-10716) was used as Ab^c^, and HRP-coupled *Neisseria gonorrhoeae* antibody (Invitrogen, PA1-73144) was used as Ab^d^. For *Staphylococcus aureus* (*S. aureus*) detection, *S. aureus* Ms mAb antibody (Abcam, ab37644) was used as Ab^c^, and HRP-coupled *S aureus* Rb pAb antibody (Abcam, ab156662) was used as Ab^d^. For methicillin-resistant *S. aureus* (MRSA) detection, Anti-Methicillin Resistant *Staphylococcus Aureus* antibody [AC10](Abcam, Ab69237) was used as Ab^c^, and HRP-coupled MRSA antibody (Abcam, ab62742) was used as Ab^d^. For *Streptococcus pneumoniae* (*S. pneumoniae*) detection, *S. pneumoniae* antibody (Invitrogen, MA1-83478) was used as Ab^c^, and horseradish peroxidase (HRP) coupled *S. pneumoniae* antibody (Invitrogen, PA1-73012) was used as Ab^d^.

#### Antibiotics

Antibiotics were purchased from Sigma Aldrich: ampicillin (A5354), gentamicin sulfate (G1914), vancomycin hydrochloride (V8138), ceftriaxone (C5793), meropenem trihydrate (M2574), clindamycin (C5269), oxacillin (28221).

#### Other

Bacto Tryptic Soy Broth (Becton Dickinson) was used as the broth for the growth of bacteria. In AST measurements, the broth:blood ratio was 1:5 by volume.

### Standard laboratory diagnosis procedure

The bacteria in the clinical blood samples were identified following a three-step process at the Clinical Microbiology Laboratory. Initially, 8–10 mL of a blood sample was drawn into a culture bottle, which contained liquid nutrient media. The bottle was placed in the *Bact Alert Blood Culture Instrument* (BioMerieux) for the detection of organism growth. The maximum time for the bottle to be held in the instrument was 5 days. If the culture was positive, a drop of the culture mixture would be put on an agar dish, which would then be incubated at 37 °C for 15–18 h. The incubated agar dish would show whether the organism was Gram-positive or Gram-negative. The final step was to identify the organism using MALDI TOF mass spectrometry (Bruker), whose operation requires several minutes.

AST was performed with a MicroScan WalkAway 96 Plus System (Beckman Coulter). Once the identification step was complete, colonies were picked off the agar plate, set up in a panel, and placed in MicroScan. The panel was incubated in MicroScan for about 15–18 h before the sensitivity was read. Some organisms needed to be held for 24 h before sensitivity was released. Therefore, the shortest TAT was 46 h (16 h culture bottle, 15 h agar plating, 15 h MicroScan).

To determine the concentration of bacteria in a clinical blood sample (colony count), 1 mL of the sample was put on a Petri dish containing tryptic soy agar (TSA) with 5% sheep blood. The dish was incubated for a 16-h at 37 °C for visual inspection and colony enumeration. To prepare samples of an isolated bacterial strain, a single colony was inoculated in growth broth and grown under aerobic conditions at 37 °C to an optical density at 600 nm (OD_600_) of 0.4–0.8. An aliquot of this culture was diluted and spiked into a known volume of phosphate-buffered saline. Samples were then serially diluted in whole human blood to obtain samples of progressively decreasing bacterial concentrations. The final concentration of samples was determined by serial dilution, plating, and colony enumeration. The colony count process was performed simultaneously with the RDAP measurements.

### The RDAP system

#### Principle of RDAP

RDAP incorporates an electrochemical sandwich immunoassay with FEED^20^, which is an ultrasensitive bio-detection technique with voltage-controlled intrinsic signal amplification. Because of its signal amplification capacity, RDAP allows direct detection of bacteria in samples without culture. A detailed description of the principle of the platform is available in previous publications^20,22,23^. Briefly, a conventional three-electrode electrochemical system is modified with an insulated gating electrode. The redox enzyme HRP is conjugated to the Ab^d^ and immobilized on the working electrode via the Ab^c^-bacterium-Ab^d^ sandwich immune complex. Electron transfer between HRP and the working electrode of the electrochemical system occurs via the entire complex^22^. The gating voltage, *V*_G_, applied between the gating electrode and the working electrode induces an electric field at the solution–enzyme–electrode interface to reduce the tunnel barrier for electrons. Therefore, the quantum tunnel current between the working electrode and HRP, which is the signal current, is amplified by *V*_G_. The signal amplification allows direct detection of bacteria in extremely low concentration (single-digit CFU/mL) blood samples without sample processing, leading to rapid, ultrasensitive, quantitative detection. The assay system features ultra-sensitivity provided by FEED and a high degree of analyte selectivity due to immunoassay. A detailed description of RDAP is given in Supplementary Note 1 in Supplementary Information.

#### Detection system and electrodes

The current prototype of RDAP consists of a set of single bacteria-specific SPEs, a gating electrode-SPE assembly, a compact potentiostat, an external power supply, and a laptop computer. SPEs were used as detection electrodes. This prototype has a LOD of 4 CFU/mL. It requires 88 min to complete a detection-ID measurement and 148 min for an AST measurement with one antibiotic. The system’s specificity was characterized using bacteria-specific SPEs to detect *E. coli*, *K. pneumoniae, P. aeruginosa, N. gonorrhoeae, S. aureus* (or methicillin-sensitive *S. aureus* (MSSA)), methicillin-resistant *S. aureus* (MRSA), and *S. pneumoniae*, which are frequent BSI causing pathogens^24^. The operation of the current RDAP prototype is based on serial measurements with single bacteria-specific SPEs. The transition to parallel measurements will require a multiplexing capacity, which will consist of an array of SPEs fabricated on a solid plate and automated parallel electrical communication between a multi-channel signal collection-processing system and the individual SPEs. The former, in principle, can be produced using microfabrication and MEMS methods and a preliminary version of the latter has been realized by one of the authors.

Commercial SPEs were purchased from Pine Research Instrumentation (RRPE1002C, Durham, NC). The working electrode (WE) is a 4 mm × 5 mm carbon electrode. The WE, the silver reference electrode (RE), and the carbon counter electrode (CE) are fabricated on the top side of the SPE. The total dimensions of the SPE are 6.1 cm × 1.5 cm × 0.036 cm. The WE was modified with a composite of carbon nanotube, Nafion, and glutaraldehyde. Briefly, a mixture of single-walled carbon nanotubes (0.2% g/mL of nanotubes in 99% dimethylformamide), Nafion (0.5 wt% in ethanol and water), and glutaraldehyde (3% in water) was deposited on the carbon WE until the composite became dried. The volume ratio of the three substances was 1:1:3. The modification procedure is described in an earlier publication^25^. To construct the detection electrode, Ab^c^ was immobilized on the modified WE by overnight incubation at 4 °C, followed by washing the electrode with de-ionized (DI) water and blocking the non-specific binding sites on the WE with bovine serum albumin.

#### Detection procedure and platform characterization

Detection was performed directly on whole blood samples at room temperature without additional sample processing or culture enrichment. The detection procedure started with the incubation of 300 µL of the sample on the WE that contained Ab^c^ on its surface, with the sample confined in a Teflon cylinder on top of the WE for 50 min at room temperature, followed by washing the WE with DI water. Then, a solution containing Ab^d^ was incubated on the WE for 30 min, followed by washing with DI. The SPE with the sandwich immune complex formed on the WE was then ready for signal detection and analysis. We have conducted an analytical validation of RDAP following the guidelines for laboratory-developed tests^26^ (see Supplementary Note 2 in Supplementary Information). The characterized items included linear range, LOD, and precision. The validation also included tests on negative clinical blood samples and positive clinical blood samples that contained a species that was different from the seven bacteria covered by the RDAP’s bacteria-specific SPEs (see Tables S1 and  S2) to ensure true negative results.

#### Detection signal analysis

**Fig. 2:**
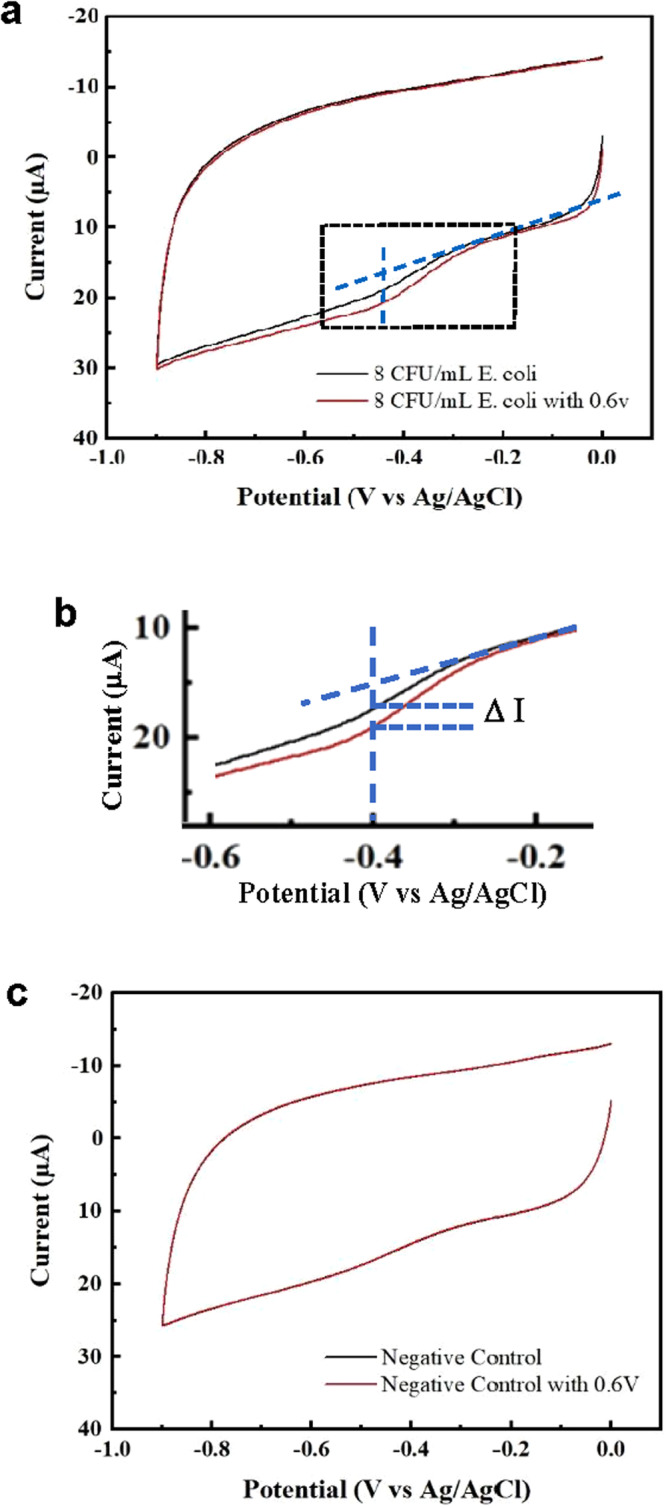
Detection signal from CVs. **a** Amplification of the detection signal from a sample with 8 CFU/mL of *E. coli*. The reduction peak (−0.4 V) current of HRP measured from the blue baseline, the dotted line, is the detection signal. **b** Focusing on the peak, the difference between the amplified peak (*V*_G_ = 0.6) and the unamplified peak (*V*_G_ = 0) is denoted Δ*I*. **c** CVs of a negative control (no bacteria) blood sample with and without *V*_G_ amplification.

Detection signals are extracted from the cyclic voltammograms (CVs) of a bacteria-specific SPE generated by the electrochemical system of RDAP, as explained in Fig. 2^27^. The black CV in Fig. 2a was obtained with an *E. coli*-specific SPE that contained an *E. coli* sandwich complex from a prepared blood sample without applying *V*_G_. The CV shows a weak HRP reduction peak near −0.4 V. The red CV was obtained with the application of *V*_G_ = 0.6 V, which amplified the peak at −0.4 V. The difference between the HRP reduction peak at −0.4 V obtained with the applied *V*_G_ (red curve) versus without V_G_ (black curve) is denoted by ∆*I* and is used as the indicator of the detection of the target bacteria (Fig. 2b). The detection signal is the current of the reduction peak of HRP (measured from the baseline) in the red CV (Fig. 2a and b). The signal’s magnitude is proportional to bacterial concentration. The signal can be amplified to a certain extent by increasing *V*_G_, leading to *V*_G_-controlled amplification of the detection signal. The absence of ∆*I* (i.e. ∆*I* = 0) indicates that the sample does not contain the target bacteria. Figure 2c shows the CVs of a bacteria-negative prepared blood sample. The CV obtained without *V*_G_ shows a step-like structure at −0.4 V, which is a characteristic of the modified working electrode. The HRP reduction peak is not observed due to the absence of the sandwich immune complex on the working electrode. Applying *V*_G_ does not cause any change to the step-like structure (i.e. ∆*I* = 0). The signal amplification allows the platform to detect the actual bacterial concentration directly in clinical blood samples without culture. Because the detection is based on the specific immuno-reaction between bacteria and their antibodies, the detection of a bacterium also identifies the bacterium in a multiple-bacteria sample, leading to simultaneous detection and ID. This process is accomplished using a set of bacteria-specific SPEs.

#### Antibiotic susceptibility testing

**Fig. 3:**
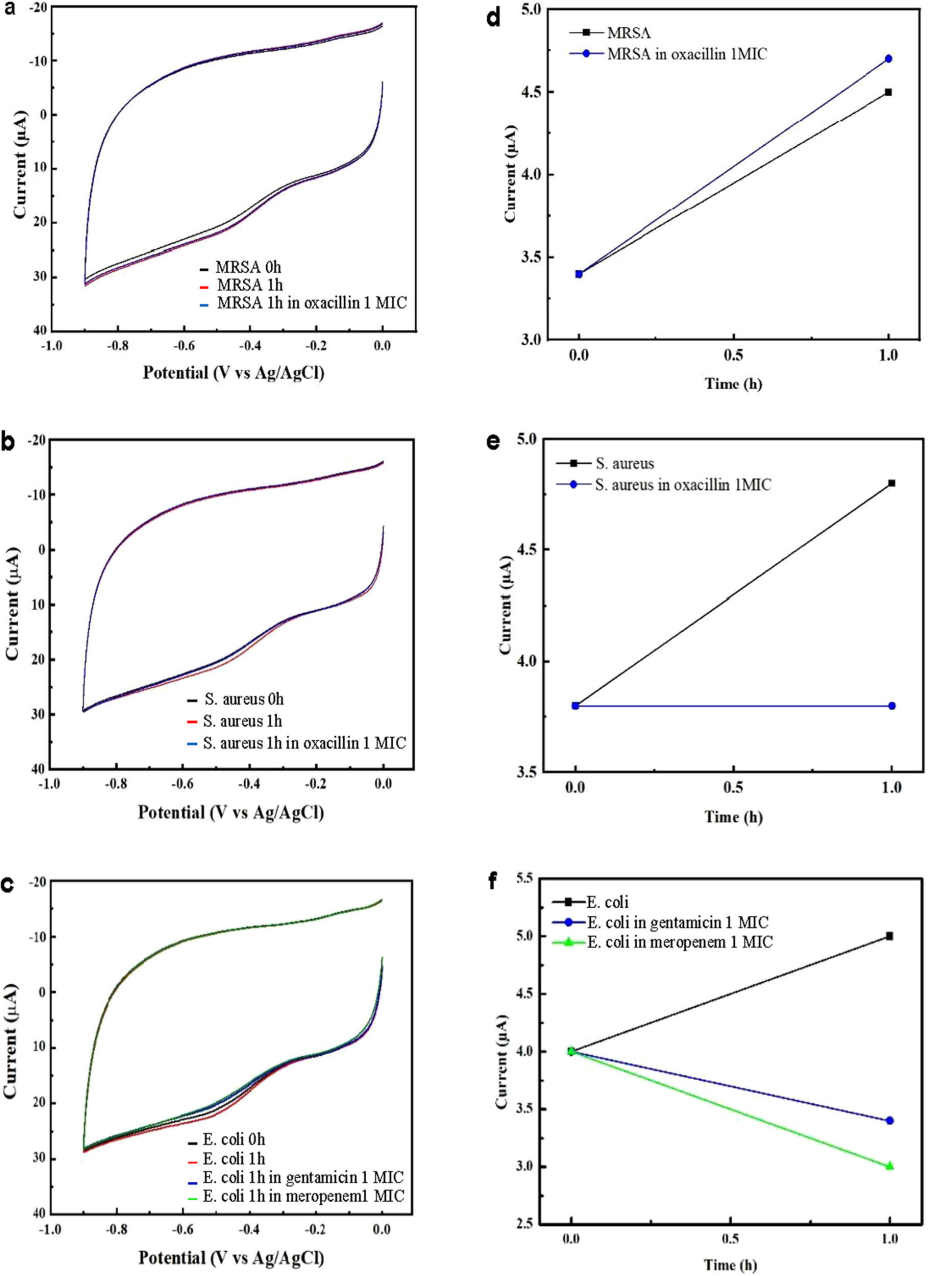
Rapid antibiotic susceptibility testing (RAST). Three types of CV responses of clinical samples containing bacteria to antibiotics over a 1-h exposure time: **a** The resistance of an MRSA strain to oxacillin, **b** The bacteriostatic response (during this short time frame) of an MSSA strain to oxacillin, and **c** The bactericidal response of an *E. coli* strain to meropenem and gentamicin. **d–f** The corresponding AIPs generated from CVs in **a–c**, respectively. All the measurements were made without bacterial isolation and standardization.

The ultrasensitive detection capability of RDAP can be used to monitor the changes in bacterial concentration in response to antibiotics over very short time frames. This capability was leveraged to achieve rapid AST (RAST), as shown in Fig. 3^28^, in which AST was performed on the sample directly without bacteria isolation and standardization, as we demonstrated previously with contrived samples^21^. RDAP performs AST by monitoring the change in the detection signal and thus bacterial concentration after exposing the bacteria in the sample to a mixture containing PBS, growth broth, and antibiotics with different concentrations for a short period of time, in this study, of 1 or 2 h. The AST result produced by RDAP, referred to as the antibiotic interaction pattern (AIP), is a plot of the detection signal versus the sample’s antibiotic exposure time. Therefore, an AIP is constructed from a set of CVs to show the change in the bacterial concentration in a sample caused by an antibiotic. AIPs show that the signal either increases, remains unchanged, or decreases during the antibiotic exposure period, indicating bacterial growth, absence of growth (bacteriostatic), or lysis of bacterial cells (bactericidal), respectively. The first case indicates resistance, and the other two cases represent different levels of susceptibility. For example, the CVs in Fig. 3a obtained from an MRSA-containing blood sample (4 CFU/mL) show the signal before (black CV) and after 1 h of exposure to the antibiotic (red CV). In the presence of oxacillin (1xMIC), the one-hour growth (blue CV) is similar to that without oxacillin because MRSA is resistant to oxacillin. On the contrary, the blue CV in Fig. 3b obtained from the MSSA-containing sample shows the absence of bacterial growth (bacteriostatic) in the presence of oxacillin, indicating that the strain is susceptible to oxacillin. In Fig. 3c, the CVs obtained from an *E. coli-*containing sample exposed to 1xMIC gentamicin and meropenem show a decrease in bacteria concentration (bactericidal), indicated the susceptibility of this strain to these two antibiotics. Figure 3d–f are the AIPs obtained from the CVs in Fig. 3a–c, respectively. Note that the black lines in Fig. 3d–f are the control AIPs obtained from samples without exposure to antibiotics.

## Results

In this study, we used RDAP to perform two types of tests on clinical blood samples. First, we performed simultaneous detection-ID (see “The RDAP system” in the “Methods” section). Second, we evaluated RAST^21^. For each type of test, we present the data for all the samples tested. For this initial clinical feasibility stage, we limited the study to only seven specific bacterial species/strains based on already developed and characterized capture/detection antibody pairs. This system may be easily adapted to the detection-identification for other species provided specific antibodies are available.

### Simultaneous detection-ID of bacterial species/strains

The simultaneous detection-ID process detects a particular bacteria/organism in the sample and indicates the detection specificity against the other bacteria, which are also the detection targets of RDAP. Seven bacteria/species-specific SPEs, namely, SPEs individually specific to *E. coli*, *K. pneumoniae*, *P. aeruginosa*, *N. gonorrhoeae*, methicillin-resistant *S. aureus* (MRSA), methicillin-sensitive *S. aureus* (MSSA) and *S. pneumoniae* were used to demonstrate simultaneous detection-ID. This range of species represents 57% of all BSIs^29^. The results are listed in Table 1. The first column shows the sample identification. The measurement data sets^30^, the RDAP cyclic voltammograms (CVs) of the bacteria-specific SPEs used for a sample, and the diagnostic results produced using MicroScan (courtesy of the Microbiology Laboratory at Saint Vincent Charity Medical Center (SVCMC)) for the same sample can be accessed via the hyperlink, Table 1, shown in ref. 30. Due to variable sample volume availability in this convenience population, not all species were evaluated for all samples. The second column shows the identity of the bacteria in the sample as determined by MicroScan and the bacterial concentration of the sample determined using standard agar plating. The remaining columns show the results of the simultaneous detection-ID of bacteria obtained via RDAP. As explained in the “Methods” section, the detection result, ∆*I*, is the difference between the HRP reduction peak currents with and without *V*_G_. In almost all cases, only the SPE specific to the target bacteria produced a non-zero ΔI. The other SPEs had Δ*I* = 0 µA, indicating the absence of a detection signal. In the cases where the Δ*I* from the off-target SPEs have non-zero values, they are conspicuously smaller than that of the Δ*I* from the target SPE. To be more specific, assuming a Δ*I* threshold cut value of 0.7 µA, the lowest true positive value, any false positive (i.e., non-zero values of Δ*I*) were always <30% of the true positive value. Of note, four of these cases represent co-detection of MRSA and MSSA. This is expected since the four antibodies used in the detection of *S. aureus* (MSSA) and MRSA were raised against whole bacteria cells rather than specific epitopes of the strains. Given that bacterial culture has reduced sensitivity for multispecies samples, it is possible that co-detection of other species/strains by our assay is not false positive but rather detection of mixed species or, in the case of MRSA and MSSA co-detection, the heterogenous population of the same species. Future work with larger dedicated sampling for reproducibility testing is required to better understand this phenomenon. The typical TAT for simultaneous detection/ID was ca. 88 min.

**Table 1:** Simultaneous detection-ID results

Sample #	Species^a^ /Bacterial concentration (CFU/mL)	Species-specific SPEΔ*I* (µA)						
		EC	KP	PA	NG	MSSA	MRSA	SP
BC2229	*EC* / 5	0.8	N/A	0	N/A	N/A	0	N/A
BC2355	*EC* / 4	1.0	0	0	0	0	N/A	0
BC2429	*EC* / 4	1.0	0	0	0	0	N/A	0
BC1422	*EC* / 4	0.7	0	0	N/A	0	N/A	0
BC1851	*KP* / 4	0	0.9	0	N/A	0	N/A	0
BC2875	*PA* / 4	0	0	1.0	N/A	0.1	0	N/A
BC1191	MSSA / 4	0	0	0	N/A	0.7	N/A	0
BC1406	MSSA / 2	0	N/A	N/A	N/A	0	0	N/A
BC2602	MSSA / 4	0	0	0	N/A	1.1	0	N/A
BC2542	MSSA / 3	0	0	0	N/A	0.7	0.2	N/A
BC2885	MSSA / 4	0	0.2	0	N/A	1.0	0	N/A
BC1009	MSSA / 4	0	0	0	0	1.0	0.2	0
BC1388	MRSA / 4	0	0	0.2	N/A	N/A	1.1	0.3
BC1715	MRSA / 4	0.2	N/A	N/A	N/A	N/A	0.8	N/A
BC2235	MRSA / 4	0	0	0	N/A	0.2	1.2	0
BC1659	MRSA / 4	0	0	N/A	N/A	0.2	1.0	N/A
Sensitivity (95% CI)		1.0 (0.49–1.0)	1.0 (0.06–1.0)	1.0 (0.06–1.0)	N/A	0.8 (0.38–0.8)	1.0 (0.49–1.0)	N/A
Specificity (95% CI)		1.0 (0.83–1.0)	1.0 (0.92–1.0)	1.0 (0.91–1.0)	N/A	1.0 (0.70–1.0)	1.0 (0.71–1.0)	N/A

*EC* *E. coli*, *KP* *K. pneumoniae*, *PA* *P. aeruginosa*, *NG* *N. gonorrhoeae*, *MSSA* methicillin-sensitive *S. aureus*, *MRSA* methicillin-resistant *S. aureus*, *SP* *S. pneumoniae*.

^a^Species identified by MicroScan.

The assay specificity of RDAP was further tested by performing simultaneous detection-ID on negative clinical blood samples, samples whose culture showed no bacterial growth, as shown in Table S1 (see Supplementary Note 3). There is an absence of a detection signal for each of the 10 samples tested. A more stringent test on the assay specificity of RDAP was conducted by performing simultaneous detection-ID on clinical samples that were determined using standard laboratory diagnosis procedure to contain a species (positive blood samples) that was different from any of the target bacteria covered by the bacteria-specific SPEs shown in Table 1. Table S2 (see Supplementary Note 3) shows the results from six clinical blood samples that met this criterion. All tests show Δ*I* = 0 µA, indicating the absence of cross-reaction and, therefore, a higher degree of specificity. This type of measurement provides specificity against the presence of potential contamination species such as *Staphylococcus epidermidis*, which is shown in Table S2.

### AST measurements

The conventional method for AST is based on the culture of bacterial isolates in standardized samples. This is a two-step process. First, bacteria colonies are spatially separated/isolated by culturing bacteria in extremely low concentrations. Then, visual inspection is used to assign the identities to the separated colonies and to transfer them into standardized solutions for the AST procedure. We have used RDAP to perform phenotypic AST measurements. In the following, we first present the AST measurements on a bacteria strain isolated from a clinical sample and spiked in a growth solution as an example to demonstrate RDAP’s capacity to perform AST on isolated bacteria as required by the conventional method. We then present the results from our RAST measurements made directly on clinical samples without bacterial isolation and standardization with the goal of further reducing TAT. As a note, for this feasibility study, we selected antibiotics from the list of antibiotics of the MicroScan in the lab to cover the resistant and susceptible cases.

**Fig. 4:**
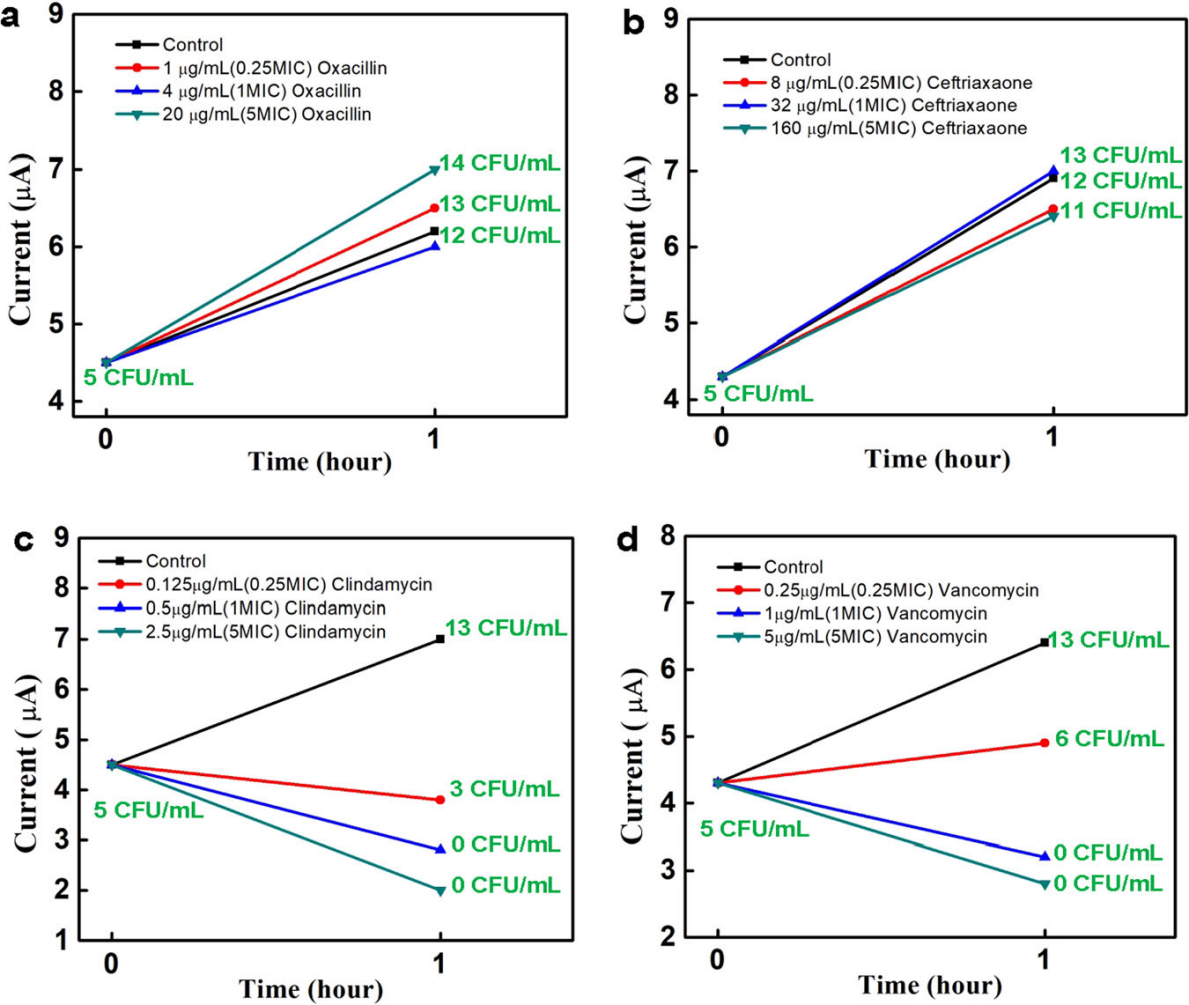
AIPs of a MRSA isolate. The AIP for the MRSA isolates from BC1722 for (**a**) oxacillin, **b** ceftriaxone, **c** clindamycin, and **d** vancomycin. The quantitative culture results shown in green are used to confirm the RDAP results.

We have isolated an MRSA strain from a clinical blood sample (BC1722) and spiked the strain into growth broth. The strain’s AST results obtained using MicroScan are shown in Table 2. The strain is resistant to ceftriaxone and oxacillin and susceptible to vancomycin and clindamycin. We exposed low-concentration (5 CFU/mL) aliquots of the strain to these specific antibiotics at different concentrations (0.25×, 1×, and 5× MIC) for 1 h before making RDAP measurements to determine bacterial concentration. Bacterial concentrations were verified by agar plating and colony enumeration. Figure 4^31^ shows the AST results of the MRSA strain as reflected by its AIPs. Figure 4a–d shows the AIPs of the strain for each antibiotic. The TAT for a single AIP was 148 min (1 h antibiotic exposure + 88 min detection with one concentration of an antibiotic). Although each antibiotic AIP was performed serially in this study, there is no fundamental reason why they cannot be performed in parallel. All results are compared to that of a control sample, which was an aliquot of the sample without antibiotics, to reflect the natural growth of the isolate.

**Table 2:** MicroScan AST of MRSA isolates from BC1722

Antibiotic	MIC (mg/L)	Interpretation^a^
Cefazolin	8	R
Cefoxitin	>4	POS
Ceftaroline	≤0.5	S
Ceftriaxone	32	R
Ciprofloxacin	>2	R
Clindamycin	0.5	S
Daptomycin	1	S
Erythromycin	>4	R
Gentamicin	≤1	S
Inducible clindamycin	≤4/0.5	NEG
Levofloxacin	4	I
Linezolid	2	S
Moxifloxacin	2	S
Oxacillin	>2	R
Rifampin	≤1	S
Synercid	0.5	S
Tetracycline	≤2	S
Trimethoprine/Sulfamethoxazone	≤0.5/9.5	S
Vancomycin	1	S

^a^R—Resistant; S—Susceptible; I—Intermediate; POS—positive; NEG—negative.

Figure 4a and b show increased bacterial concentration or growth in the aliquots that have been exposed to oxacillin and ceftriaxone, respectively. The results confirm the resistance of this isolate to these antibiotics and are consistent with the MicroScan results (Table 2). Although the signal for the 5x MIC treatment is higher, this represents a single CFU/mL difference and, therefore, only a small difference in cell concentration with respect to the doubling rate of bacteria. Figure 4c and d show that bacterial growth is suppressed by clindamycin and vancomycin, respectively, reflecting the susceptibility of this isolate to these antibiotics. Furthermore, RDAP showed that the degree of growth inhibition scales with the concentrations of the antibiotics, as shown in Fig. 4c, d. Therefore, the platform’s AST results agree with those obtained using MicroScan. However, the platform produced the results 15 h faster. Table 3 is a summary of the AST results obtained using RDAP on this isolated strain. Antibiotic susceptibility was based on the value of δ*I* (see Table 3), which is the difference between the detection signal current (Δ*I*) from an antibiotic-exposed sample at the end of the antibiotic exposure time and that at the start without antibiotics. Specifically, based on our prior assay development work^21^ we defined susceptibility (S) as δ*I* < 0.25 µA, intermediate resistance (I) as 0.25 ≤ δ*I* ≤ 0.5 µA, and resistance (R) as δ*I* > 0.5 µA. The results agree with those produced by MicroScan. Our intention in presenting the AST results on an isolated strain is to show the capacity of RDAP to achieve similar AST results provided by standard technologies. In Table 3 and the table that follows Table 4 and Table 5, certain antibiotics have different RDAP interpretations according to the concentration. In these cases, the bacteria is called resistant if any concentration of antibiotic at or below the MIC had δ*I* > 0.5 µA. AST on an isolated strain was also demonstrated in our previous publication^21^.

**Table 3:** Isolate AST from RDAP

Antibiotic	Concentration (× MIC)	MicroScan MIC (mg/L)	MicroScan interpretation	δ*I* (µA)	RDAP interpretation
BC1722 (MRSA)					
Oxacillin	0.25	>2	R	1.9	R
	1			1.3	R
	5			2.5	R
Ceftriaxone	0.25	32	R	2.1	R
	1			2.7	R
	5			2.0	R
Clindamycin	0.25	0.5	S	−0.7	S
	1			−1.7	S
	5			−2.5	S
Vancomycin	0.25	1	S	0.5	I
	1			−1.1	S
	5			−1.6	S

R—Resistant; S—Susceptible; I—Intermediate.

**Table 4:** MicroScan AST of an *E. coli* strain in BC2229

Antibiotic	MIC (mg/L)	Interpretation
Amikacin	≤16	S
Amoxicillin/K Clav	8/4	S
Ampicillin/Sulbactam	>16/8	R
Ampicillin	>16	R
Aztreonam	≤4	S
Cefazolin	4	S
Cefepime	≤2	S
Cefotaxime	≤2	S
Cefotaxime/K Clav	≤0.5	
Cefotaxime-ESBL	≤1	
Ceftazidime	≤1	S
Ceftazidime/K Clav	≤0.25	
Ceftriaxone	≤1	S
Cefuroxime	≤4	S
Ciprofloxacin	≤1	S
Ertapenem	≤0.5	S
Gentamicin	>8	R
Imipenem	≤0.5	S
Levofloxacin	0.5	S
Meropenem	≤1	S
Piperacillin/Tazobactam	≤4	S
Tetracycline	>8	R
Ticarcillin/K Clav	≤8	S
Tigecycline	≤2	S
Tobramycin	>8	R
Trimethoprine/Sulfamethoxazone	>2/38	R

R—resistant; S—susceptible.

**Table 5:** RAST results from clinical blood samples

Sample # (species^a^, CFU/mL)	Antibiotic	Concentration (×MIC)	MicroScan MIC (mg/L)	MicroScan interpretation	δ*I* (µA)/Exposure time(h)	RDAP interpretation
BC0035 (*EC*, 4)	Ampicillin	1 MIC	>16	R	1.1 / 2	R
	Gentamicin	1 MIC	2	S	0 / 2	S
BC0633 (*EC*, 4)	Ampicillin	1 MIC	>16	R	1.0 / 2	R
		2 MIC			1.0 / 2	R
BC2377 (*EC*, 3)	Ampicillin	1 MIC	<8	S	0.2 / 1	S
	Ceftriaxone	1 MIC	<1	S	0.2 / 1	S
	Gentamicin	1 MIC	<1	S	0.2 / 1	S
BC2002 (*EC*, 4)	Ampicillin	1 MIC	<8	S	−0.5 / 1	S
BC2229 (*EC*, 4)	Gentamicin	1 MIC	<1	S	−0.8 /1	S
	Meropenem	1 MIC	<1	S	−1.3 / 1	S
*BC0250* (*EC*, 4)	Ampicillin	1 MIC	>16	R	1.7 / 2	R
	Ceftriaxone	1 MIC	<1	S	0.2 / 2	S
BC3823 (*KP*, 4)	Ceftriaxone	1 MIC	<8	S	−0.6 / 2	S
	Meropenem	1 MIC	<4	S	0.9 / 2	R
BC2352 (*KP*, 4)	Gentamicin	1 MIC	2	S	0.2 / 1	S
	Meropenem	1 MIC	<1	S	0 / 1	S
BC0763 (MSSA, 4)	Oxacillin	1 MIC	0.5	S	0 / 1	S
BC1815 (MSSA, 4)	Clindamycin	1 MIC	0.5	R	0.8 / 2	R
	Oxacillin	1 MIC	<0.25	S	−0.1 / 2	S
BC1839 (MSSA, 4)	Oxacillin	0.5 MIC	1	S	−0.4 / 1	S
		1 MIC			−0.4 / 1	S
BC1845 (MSSA, 4)	Oxacillin	1 MIC	0.5	S	−0.7 / 1	S
		2 MIC			−0.6 / 1	S
BC1902 (MSSA, 5)	Oxacillin	3 MIC	0.5	S	−0.8 / 1	S
BC2092 (MSSA, 4)	Oxacillin	1 MIC	0.5	S	−0.3 / 1	S
BC2950 (MSSA, 4)	Clindamycin	1 MIC	0.5	R	1.3 / 2	R
	Oxacillin	1 MIC	<0.25	S	0 / 2	S
BC2271 (MSSA, 4)	Clindamycin	1 MIC 3 MIC	<0.25	S	0 / 1 −0.1 / 1	S S
BC0482 (MSSA, 4)	Oxacillin	1 MIC	0.5	S	0 / 2	S
BC0186 (MRSA, 4)	Oxacillin	1 MIC	>2	R	1.2 / 2	R
	Clindamycin	1 MIC	<0.25	S	0.2 / 2	S
BC0317 (MRSA, 3)	Oxacillin	0.3MIC	>2	R	0.5 / 2	I
		1 MIC			0.5 / 2	I
		2 MIC			0.5 / 2	I
	Vancomycin	0.3 MIC	1	S	1.3 / 2	S
		1 MIC			0.5 / 2	I
		2 MIC			0.6 / 2	I
BC1585 (MRSA, 4)	Ceftriaxone	1 MIC	16	R	2.0 / 1	R
	Vancomycin	1 MIC	1	S	0 / 1	S
		2 MIC			0.1 / 1	S
BC0323 (MRSA, 4)	Clindamycin	0.5 MIC	<0.25	R	0.4 / 2	I
		1 MIC			0.6 / 2	R
		2 MIC			0.39 / 2	I
BC0392 (MRSA, 4)	Oxacillin	1 MIC	>2	R	1.7 / 2	R
	Gentamicin	1 MIC	<1	S	−1.5 / 2	S
BC0432 (MRSA, 4)	Clindamycin	0.5 MIC	<0.25	R	1.2 / 2	R
		1 MIC			1.8 / 2	R
		2 MIC			2.0 / 2	R
BC0881 (MRSA, 4)	Oxacillin	1 MIC	>2	R	0.9 / 1	R
	Gentamicin	1 MIC	<1	S	0 / 1	S
BC1263 (MRSA, 4)	Ceftriaxone	1 MIC	>32	R	2.2 / 2	R
	Vancomycin	1 MIC	1	S	0.2 / 2	S
BC1540 (MRSA, 4)	Ceftriaxone	1 MIC	16	R	2.4 / 2	R
	Vancomycin	1 MIC	1	S	0.1 / 2	S

^a^Species identified by MicroScan: *EC E. coli*, *KP K. pneumoniae*, *MSSA methicillin-sensitive S. aureus*, *MRSA methicillin-resistant S. aureus*.

R—Resistant; S—Susceptible; I—Intermediate.

**Fig. 5:**
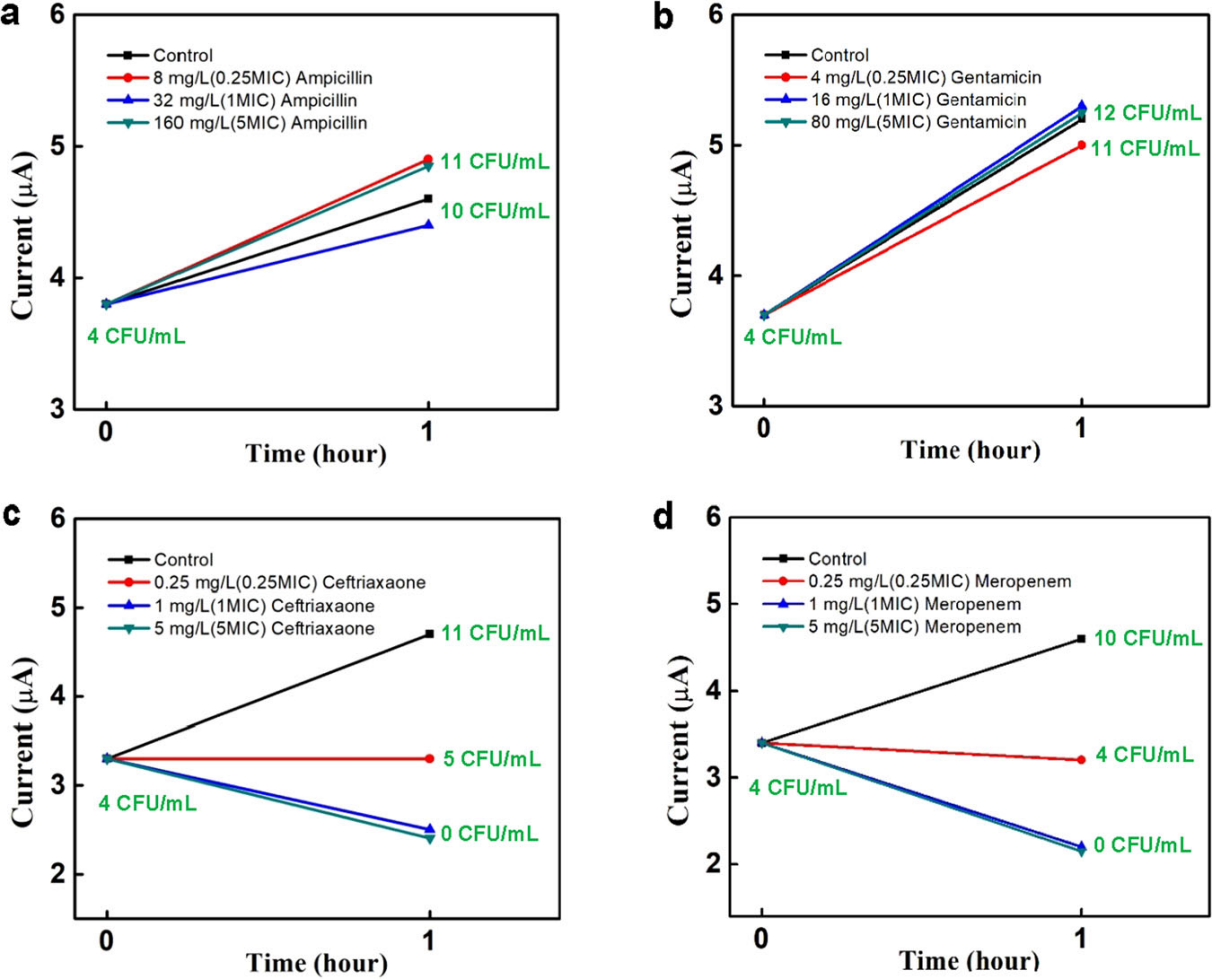
AIPs from RAST of a clinical sample. AIPs obtained using RDAP on unprocessed clinical sample (BC2229) containing *E. coli* (4 CFU/mL) exposed to (**a**) ampicillin, **b** gentamicin, **c** ceftriaxone, and **d** meropenem. The quantitative culture results shown in green are used to confirm the RDAP results.

Following the same procedure as shown in the example above, we used RDAP to perform AST on unprocessed clinical whole blood samples without bacterial isolation and standardization. This direct approach to AST, RAST, further shortens the TAT. Table 4 shows the Microscan AST results of a clinical blood sample (BC2229) containing 4 CFU/mL of *E. coli*, determined by agar plating. This strain is resistant to ampicillin and gentamicin and susceptible to ceftriaxone and meropenem. Figure 5 shows the AIPs for these antibiotics on this strain via RAST, confirming the susceptibility to ceftriaxone and meropenem and resistance to ampicillin and gentamicin. As in the isolate-based AST, the antibiotic exposure time for RAST on this sample was only 1 h. Again, the TAT was 148 min for each AIP. RAST was performed on 27 independent clinical samples, including BC-2229 (see Table 5). RAST shortened the TAT by eliminating the isolation and standardization processes.

In Table 5^32^, the first column shows the sample identification. The AIPs of a sample and the diagnostic results produced using MicroScan (courtesy of the Microbiology Laboratory at SVCMC) for the same sample can be accessed via the hyperlink, Table 5, shown in ref. 32. Also shown in the first column are the species identity (MicroScan) and bacterial concentration (agar plating). The remaining columns from left to right show the antibiotics used in the AST measurements, their concentrations relative to the MIC value, the MIC value of the antibiotic, the interpretation of the AST results provided by MicroScan, the value of δ*I*, and the AST interpretation provided by RDAP based on δ*I* as described above. As described in the sections “Methods” and “Discussion”, δ*I* is used as an indicator of the category of AST results.

## Discussion

Numerous studies have shown that the indiscriminate administration of broad-spectrum antibiotics is one of the main contributors to the increasing prevalence of antibiotic resistance^33,34^. On the other hand, delayed administration of antibiotics leads to increased mortality in BSI and sepsis^8,10^. This is further reinforced by governmental standards of quality and reimbursement^35,36^. Aligning the competing interests of antibiotic stewardship to reduce the prevalence of antibiotic resistance, timely administration of antibiotics for life-threatening BSI requires rapid, sensitive diagnostics that are not yet clinically available. There is an immediate unmet need for culture-free, ultrasensitive assays capable of providing rapid diagnosis of BSI. Here, we demonstrate the feasibility of RDAP for near real-time diagnosis of BSI, including AST. This represents a significant advancement of our platform from the previous work with contrived samples^21^ to clinical samples.

RDAP was able to detect and identify the correct species (as determined by standard lab procedure) in 15 out of 16 samples (93.8% accuracy). The one sample (BC1406 in Table 1) that was not detected had a bacterial concentration of only 2 CFU/mL, which was below our platform’s current LOD of 4 CFU/mL. Table 1 shows cross-reaction in the detection of MSSA vs MRSA (BC2542, BC1009, BC2235, and BC1659). This is not surprising as this is a strain level (rather than species level) distinction and may represent the heteroresistance of *S. aureus* in the sample. In the detection of one strain, RDAP shows a non-zero Δ*I* for the other strain. However, in all such cases, Δ*I* was significantly less (<30%) for the off-target strain than for the target strain. In this case, though, the sample’s oxacillin susceptibility result from AST can be used to differentiate MRSA from MSSA. The observed cross-reactivity was caused by the nature of the antibodies used for the detection of the two strains. The four antibodies used in the detection processes were raised against whole bacteria cells (see the subsection “Reagents” in the “Methods” section) rather than specific epitopes of the bacteria. Since MRSA is evolved from MSSA, the observed cross-reactivity was expected. This issue could be, in principle, solved by using more specific antibodies. For example, the commercial rabbit anti-PBP2a antibody would be used as the detection antibody to replace the current anti-MRSA antibody, that was raised against MRSA bacteria cells. Further, the anti-protein A antibody could be used to detect MSSA. There were also cross-reactivity events at the species level (BC2875, BC2885, BC1715 and BC1388). Again, the Δ*I* was significantly less (<27%) for the off-target species than for the target species. Therefore, the species can still be identified even in the presence of cross-reactivity. In the case where the cross-reactivity cannot be completely eliminated, larger studies could be performed to define a cut point for Δ*I* to define positivity.

Several cases in Table 1 show a large detection signal and a much smaller signal from a different detection electrode, suggesting the possibility of the detection of mixed organisms, although the gold standard, culture, and MicroScan, showed only one organism was present. MicroScan performs ID and AST after the blood culture is positive. Therefore, slow-growing or low-concentration species may not grow sufficiently to be isolated and provided as input to the MicroScan assay. The sensitivity of Microscan depends as much on the input (i.e., initial culture) as it does on the analytics of the assay itself. On the contrary, the RDAP assay works directly on the original sample and therefore removes the potential bias toward high concentration rapidly growing species provided by the culture step. It is possible that the low value of other organisms may represent true positives that are missed by the current gold standard and is one potential interpretation of those readings. However, culture remains the gold standard in clinical microbiology. Therefore, differentiating between background and true positives, as suggested, would require extensive clinical record data and physician adjudication to evaluate the potential for a multispecies infection. This data was not available for this study.

When using RDAP to perform AST, the assignment of a species to the resistant (R), intermediate (I) and susceptible (S) categories with respect to an antibiotic was defined a priori as δ*I* < 0.25 µA—susceptible; 0.25 ≤ δ*I* ≤ 0.5 µA—intermediate resistance; δ*I* > 0.5 µA—resistant. These initial thresholds were determined based on our prior work using contrived samples^21^. The interpretative standards for MICs were provided by MicroScan as per standard CLSI breakpoints. Using this metric, RDAP correctly identified antibiotic susceptibility (as determined by MicroScan) in 38 out of 42 sample:antibiotic pairs (90.5%). However, if the interpretation is binarized such that intermediate resistance is considered resistance, then the performance improved to 95.4% (40 out of 42) accuracy. This is because two pairs were labeled as resistant according to MicroScan but intermediate according to RDAP (i.e., BC0317 & BC0323). This makes sense clinically, as antibiotics with intermediate resistance would typically be avoided, similar to those with resistance. That leaves two discordant predictions. Specifically, one pair (BC3823) was identified as resistant to meropenem when MicroScan was labeled as sensitive, and one (BC0371) was identified as resistant to vancomycin when MicroScan was labeled as sensitive. In both cases, RDAP makes the more conservative call of resistance rather than sensitivity. However, since the δI thresholds were determined a priori, future refining of the definition of susceptibility based on δI could improve diagnostic performance. This would require a larger population-based study akin to those used to adjust MIC-based breakpoints for standard AST. An unexpected finding in this study was that the interpretation (based on δ*I*) seemed independent of the antibiotic concentration (relative to the published MIC breakpoint for that species) used. In the 9 cases where multiple concentrations of an antibiotic were considered on the same isolate, the interpretations were the same for each concentration. This is assuming the interpretation is binarized such that intermediate resistance is considered resistant. The detection of resistance at 0.5MIC for samples BC0323 and BC0432 could represent inducible or hetero-resistance. Notably, this is a small subset, and future studies are required to standardize the concentrations of antibiotics used for RDAP. That study should be guided by current published MIC breakpoints.

Although the potential clinical utility of RDAP for culture-free diagnosis of BSI has been demonstrated, there are still several limitations and caveats of the platform in its current form. The results presented here are based on serial measurements. To fully exploit the culture-free approach, multiplexing capacity will be needed to make measurements on several samples simultaneously (i.e., parallel measurements). With multiplexing, the analytic time of the detection-ID and AST steps will remain at 88 and 148 min, respectively. The TAT of RDAP represents a substantial potential improvement over standard commercial diagnostic technologies such as FilmArray, LightCycler, and AcceleratePheno, which require a positive culture of samples as their input. Therefore, they suffer the underlying sensitivity, specificity and TAT issues associated with culture. The LOD of the current prototype is ~4 CFU/mL. However, this is fundamentally dependent on the sample volume applied to the SPE. Therefore, the LOD could be improved by increasing the sample volume applied. Currently, this volume is 300 µL. For the 75 measurements of a sample for the detection-ID-AST diagnosis process (15 ID targets with 5 AST each), the required sample volume will be 22.5 mL, which is comparable to the typical volume of blood required for standard culture (i.e., 10–30 mL). So far, only seven species (3 Gram-positive and 4 Gram-negative) have been considered. The number of bacteria-specific SPEs with antibody pairs will need to be expanded to include many more pathogens to achieve 95% of all BSIs. As noted above, the current AST is based on MIC breakpoints that require overnight culture. Adoption of RDAP will require establishing a new paradigm for breakpoints based on δI. The present study was not a direct comparison of time to result. Specifically, blood samples were stored and analyzed by RDAP after results from standard blood cultures and MicroScan were available. Future clinical studies will require real-time collection of independent samples for direct time-to-result comparison. This study was limited to a single small institution with a small sample size. True evaluation of sensitivity and specificity in comparison to blood culture will require larger multi-center studies, given the low prevalence of bacteremia among blood culture samples (<10%). The RDAP in its present form does not include an internal control for the authentication of negative results, although RDAP has been tested with blank blood (negative control) samples (see Supplementary Note 2 in Supplementary Information) to ensure negative results. The advanced prototype we are currently developing will include an internal control channel to perform simultaneous tests on negative control for each sample. Lastly, in this pilot study, *E. coli* was used as a model organism for the analytical validation of the RDAP. In our future studies, the validation process will include all seven organisms in contrived and clinical samples.

Based on the results from the cohort of clinical blood samples used in this clinical feasibility study, we conclude that RDAP can (1) perform simultaneous detection-ID and AST on clinical samples containing common BSI-causing pathogens without culture enrichment; (2) achieve rapid and accurate phenotypic AST with 1-h antibiotic exposure; and (3) perform AST directly from a blood sample without prior species isolation. These features have the potential to dramatically reduce TAT and subsequently provide actionable results to clinicians, allowing the use of narrow-spectrum antibiotics before the second dose of empiric broad-spectrum antibiotics is given. Currently, RDAP needs 88 min to perform the detection-ID step. Considering the 16-plus hours required by the standard lab procedure for this step, RDAP is already a near real-time diagnostic for the screening of specific species, e.g. MRSA, which is a test frequently ordered by clinicians. It is likely that the sample incubation time and the antibiotic exposure time can be further reduced so that the serial execution of the 3-step diagnosis will also be within the near real-time time scale^37^. In this feasibility study, we present the diagnosis results from a small cohort of clinical samples to demonstrate a possible paradigm change in the administration of antibiotics and to introduce a new direction to solve a critical medical issue that threatens public health.

### Reporting summary

Further information on research design is available in the Nature Portfolio Reporting Summary linked to this article.

### Supplementary information

Supplemental Information

Reporting Summary

## Data Availability

The numerical data used to plot Fig. 2 can be accessed at 10.6084/m9.figshare.25432729.v1 and ref. 27. The numerical data used to plot Fig. 3 can be accessed at 10.6084/m9.figshare.25432732.v1 and ref. 28. The numerical data used to plot Fig. 4 can be accessed at 10.6084/m9.figshare.25432735.v1 and ref. 31. The source data used to compile Table 1 can be accessed at 10.6084/m9.figshare.25322557.v1 and ref. 30. The source data used to compile Table 5 can be accessed at 10.6084/m9.figshare.25322749.v2 and ref. 32.
