# Supplementary material for: Rapid diagnosis of bloodstream infections using a culture-free phenotypic platform
Source: Commun Med (Lond). 2024 Apr 23;4:77. doi: 10.1038/s43856-024-00487-x (PMC11039749; doi:10.1038/s43856-024-00487-x)
Supplement: Supplementary file 1 — Supplemental Information [file 43856_2024_487_MOESM1_ESM.pdf]

Supplementary information

Rapid diagnosis of bloodstream infections using a culture-free phenotypic platform

Xuyang Shi[1], Shivani Sharma[2], Richard A. Chmielewski[3], Mario J. Markovic[4], J. Scott VanEpps[5,6,7] & Siu-Tung Yau[1,2,8],

1. Department of Electrical Engineering and Computer Science, Cleveland State University, Cleveland, Ohio, USA.
2. Rapidect Inc., Solon, Ohio, USA
3. Saint Vincent Charity Medical Center, Cleveland, Ohio, USA.
4. Department of Laboratory Medicine, Saint Vincent Charity Medical Center, Cleveland, Ohio, USA.
5. Department of Emergency Medicine, University of Michigan, Ann Arbor, Michigan, USA.
6. Weil Institute for Critical Care Research and Innovation, University of Michigan, Ann Arbor, Michigan, USA.
7. Biointerfaces Institute, University of Michigan, Ann Arbor, Michigan, USA.
8. The Applied Bioengineering Program, Cleveland State University, Cleveland, Ohio, USA.

Correspondence author J.S.V. (email: jvane@med.umich.edu); S.-T.Y. (email: s.yau@csuohio.edu)

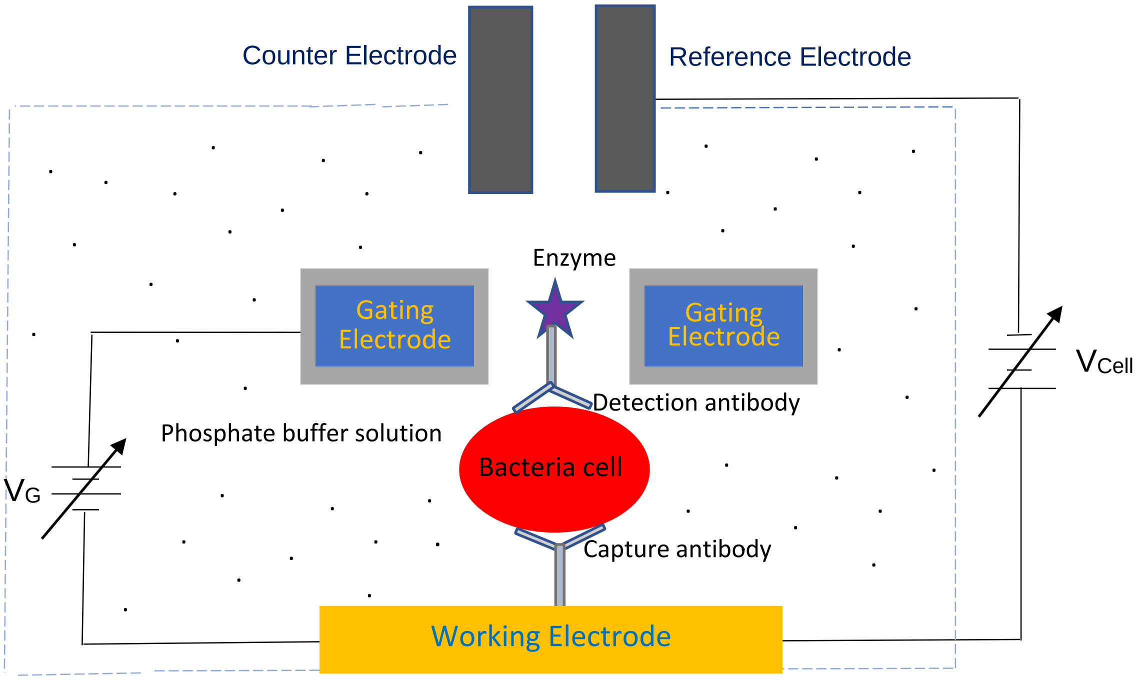

Figure S1 Schematic description of the RDAP system.

Supplementary Note 1 - RDAP system

Field effect enzymatic detection (FEED)[1] is the basic detection mechanism of RDAP. The FEED system consists of a conventional three-electrode electrochemical cell with a cell potential V_{cell} connected between the working electrode (WE) and the reference electrode. A redox enzyme used as the sensing element of FEED is immobilized on the WE. The isolation of the enzyme's active site by a polypeptide network leads to reduced electron transfer between the enzyme and WE, creating a fundamental limit to the sensitivity and therefore the limit of detection for amperometric biosensors[2]. The cell contains an electrolytic solution. An electric double layer is present near the surface of WE. The cell is modified with an insulated gating electrode for applying a gating voltage V_G between the gating electrode and the WE, upon which the redox enzyme is immobilized. V_G modifies the interfacial charge distribution. When V_G is positive, additional negative charges are induced on WE and additional positive ions

from the solution are induced at the solution-enzyme-electrode interface. Some positive ions are able to set up electric fields within the polypeptide network in the presence of the transferring electrons residing at the enzyme's active sites. Quantum tunneling of electrons from the active site through the polypeptide barrier to WE gives rise to the signal current[2]. The energy barrier can be lowered by the electric field so that the tunneling rate is enhanced[3,4]. The result of this process is an amplified signal current, an intrinsic property of the system. Figure S1 shows the components of the FEED system with the exception that the redox enzyme is not directly immobilized on the working electrode.

RDAP incorporates FEED with the immuno-sensing methodology, whose most essential component is the immune complex that is immobilized on the working electrode. Figure S1 shows the capture antibody-bacteria-detection antibody (Ab^c-bacteria-Ab^d) sandwich immune complex, in which the enzyme used as the sensing element is conjugated to Ab^d. The enzyme immobilized on the WE of FEED is replaced by the sandwich immune complex. In addition to the intrinsic signal amplification provided by FEED, RDAP also features a mediator-less operation: the transfer of electrons between the enzyme and WE occurs through the complex without using diffusive mediators[5].

Supplementary Note 2 - Analytical validation

We have conducted an analytical validation of RDAP following the guidelines for laboratory-developed tests[6]. The validation was performed with contrived *E. coli* blood samples. The linear range was found to be 4-3000 CFU/mL (10 concentrations; 3 replicates on same day). The LOD (4 CFU/mL) of RDAP was empirically determined by testing serial dilutions of contrived blood samples with a known concentration of *E. coli* in the linear range (4 concentrations near the low end of range; 8 replicates over 5 days). Three concentrations, 4, 50, 1000 CFU/mL, that cover the low, intermediate and high concentration ranges were used to indicate the precision of the platform.

The 95% confidence interval (95%CI) is a measure of the precision of an assay. At 4 CFU/mL, where the RDAP is intended to be used for BSIs, the platform yields a 95% CI value of 3.58 – 4.20 CFU/mL (based on 3 replicates over 5 days), indicating a high level of precision. Additionally, RDAP has been tested with 20-30 blank blood samples (negative control) to ensure its ability to produce true negative results.

Supplementary Note 3 - Additional specificity tests

The assay specificity of RDAP was further tested by performing simultaneous detection-ID on negative clinical blood samples, whose culture showed no bacterial growth. The results are shown in **Supplementary Table S1**, which indicates the absence of detection signal ΔI.

Supplementary Table S1. Negative Blood Samples

Sample #	Species-specific SPE ΔI (μA)					
	EC	*KP*	*NG*	*SP*	*SA*	*MRSA*
BC1505	0	0	0	0	0	0
BC1501	0	0	0	0	0	0
BC1499	0	0	0	0	0	0
BC1497	0	0	0	0	0	0
BC1495	0	0	0	0	0	0
BC1493	0	0	0	0	0	0
BC1489	0	0	0	0	0	0
BC1487	0	0	0	0	0	0
BC1467	0	0	0	0	0	0
BC1503	0	0	0	0	0	0

As a more stringent test of specificity, simultaneous detection-ID measurements were performed on clinical blood samples that were determined using standard laboratory diagnosis procedure to contain a bacterial species that was different from the set of species covered by the bacteria-specific SPEs shown in **Table 1**. **Supplementary Table S2** shows the results from 6 such clinical blood samples. All tests show no detection signals (ΔI=0), indicating the absence of cross

reaction and therefore a high degree of specificity. The data sets of **Table S1** and **Table S2**[7], the RDAP cyclic voltammograms (CVs) of the bacteria-specific SPEs used for a sample, and the diagnostic results produced using MicroScan (courtesy of the Microbiology Laboratory at Saint Vincent Charity Medical Center (SVCMC)) for the same sample can be accessed via the hyperlinks, **Table S1** and **Table S2**, shown in reference 7.

Supplementary Table S2. Specificity Tests on Additional Positive Blood Samples

Sample #	Species	Species-specific SPE ΔI (μA)					
		EC	*KP*	*PA*	*NG*	*MRSA*	*SA*
BC1482	*S. Epidermidis*	0	0	0	0	0	0
BC1471	*E. cloacae complex*	0	0	0	0	0	0
BC1407	*P. mirabilis*	0	0	0	0	0	0
BC1250	*S. Epidermidis*	0	0	0	0	0	0
BC1207	*A. baum/nosoc grp*	0	0	0	0	0	0
BC2269	*E. faecium*	0	0	0	0	0	0

References

1. Choi, Y. & Yau, S.-T. A Field-Effect Enzymatic Amplifying Detector with Pico-Molar Detection Limit. *Analytical Chemistry* **81**, 7123-7126 (2009).

2. Csoregi, E., Gaspar, S., Niculescu, M., Mattiasson, B. & Schuhmann, W. (eds. Cuyper, M.D. & Bulte, J.W.M.) 105 (Kluwer Academic Publishers, Dordrecht, Boston, London, 2001).

3. Tans, S. J., Verschueren, A. R. M. & Dekker, C. Room-temperature transistor based on a single carbon nanotube. *Nature* **393**, 49-52 (1998).

4. Yau, S.-T. & Qian, G. A prototype protein field-effect transistor. *Applied Physics Letters* **86**, 103508 (2005).

5. Wang, J., Xu, Y. & Yau, S.-T. Mediatorless Immunoassay with Voltage-Controlled Intrinsic Amplification for Ultrasensitive and Rapid Detection of Microorganism Pathogens. *ChemElectroChem* **1**, 741 –746 (2014).

6. Burd, E. M. Validation of Laboratory-Developed Molecular Assays for Infectious Diseases. *Clinical Microbiology Rev*iew **23**, 550–576 (2010).

7. The data sets for Table S1 and Table S2.
